# Editorial

**Published:** 1875-12

**Authors:** 


### Editorial.

This issue of the combined Journal and Examiner closes the volumes of both these journals for the year 1875. Beginning the year as competitors for professional favor and patronage, and honorable rivals in the promotion of scientific progress and harmony among medical men, it is quite seemly and suggestive that these two medical periodicals should finish their annual contribution to our store of knowledge united, not only in purpose, but in fact.

Standing as we now do, at the end of the year, in the places of our predecessors, it becomes our duty—in which we take great pleasure—to thank for them the many contributors and subscribers who sustained the two journals before we became connected with them; and, for ourselves, we would cordially express our very grateful obligations for their material assistance during the last three months.

We are stimulated by the memory of the past kindness of our good friends to renewed efforts and more industrious exertions to make ourselves worthy of their good will and kind acts in the future.

The January number will, as usual, be issued promptly, and in a new dress and style.

It will be enlarged by the addition of another form of sixteen pages to the present size, summing up, in all, ninety-six pages; making at the end of the year a volume of eleven hundred and fifty-two pages. Its capacity will be still further increased by reducing the type of thirty-two pages to brevier.

These two changes will be equivalent to the addition of about twenty pages such as we now have.

While the publishers have thus liberally ventured upon the internal improvement of the Journal and Examiner, they intend to spare no pains or expense in the mechanical execution of the work connected with its publication, to make it attractiye in appearance.

The January number will be sent to all the old subscribers of the CHICAGO MEDICAL JOURNAL and MEDICAL EXAMINER, with the hope that they will renew their subscriptions and send the names of many of their friends as new subscribers.

THE HOMŒOPATHIC COLLEGE OF THE UNIVERSITY OF MICHIGAN.—The University has begun its annual fall session of study in its various departments with a new school added to its facilities of instruction—that designated the Homœopathic College. This college consists —in its faculty—of two professors, who are supposed to represent the peculiar tenets of its peculiar *pathy*; one teaches therapeutics, the other, practice of medicine. The building of the new college being situated on the University grounds, the instruction in the remaining branches of a medical education is received in the old department of medicine and surgery. Here the students of the new college, with the regular students of the elder department, are taught in anatomy, physiology, chemistry, surgery and obstetrics, and with them, at the close of each term, are examined by the teachers of these branches, and receive with them a uniform certificate of attendance and proficiency.

The students of this young college who carry away the diploma of the University may justly congratulate themselves on having had for their instructors such illustrious teachers as the venerable Professors Ford and Douglas, Professors MacLean and Dunster. Whether these professors may congratulate themselves on being teachers to the new college, is another matter.

APPLES OF GOLD IN PICTURES OF SILVER.—"See how intimately, how indissolubly that part of medicine which takes for its base the particular study of the generative system in woman, is linked with the disorders of the alimentary, vascular and nervous systems: that is, a pure specialty cannot exist, a more monstrous thing cannot be conceived."—*Robert Barnes.*

